# Circulation derived from 4D flow MRI correlates with right ventricular dysfunction in patients with tetralogy of Fallot

**DOI:** 10.1038/s41598-021-91125-2

**Published:** 2021-06-02

**Authors:** Nanae Tsuchiya, Michinobu Nagao, Yumi Shiina, Shohei Miyazaki, Kei Inai, Sadayuki Murayama, Shuji Sakai

**Affiliations:** 1 Department of Radiology, Graduate School of Medical Science, University of the Ryukyus, Okinawa, Japan; 2 Department of Diagnostic Imaging and Nuclear Medicine, Tokyo Women’s Medical University, 8-1 Wakamatsu Kawada, Tokyo, 1628666 Japan; 3 Department of Pediatric Cardiology, Division of Clinical Research for ACHD, Tokyo Women’s Medical, Tokyo, Japan; 4 Cardiovascular Center, St. Luke’s International Hospital, Tokyo, Japan; 5 Cardio Flow Design Inc., Tokyo, Japan; 6 Department of Pediatric Cardiology, Division of Clinical Research for Adult Congenital Heart Disease, Tokyo Women’s Medical University, Tokyo, Japan

**Keywords:** Cardiology, Diseases

## Abstract

We used 4D-flow MRI to investigate circulation, an area integral of vorticity, in the main pulmonary artery (MPA) as a new hemodynamic parameter for assessing patients with a repaired Tetralogy of Fallot (TOF). We evaluated the relationship between circulation, right ventricular (RV) function and the pulmonary regurgitant fraction (PRF). Twenty patients with a repaired TOF underwent cardiac MRI. Flow-sensitive 3D-gradient sequences were used to obtain 4D-flow images. Vortex formation in the MPA was visualized, with short-axis and longitudinal vorticities calculated by software specialized for 4D flow. The RV indexed end-diastolic/end-systolic volumes (RVEDVi/RVESVi) and RV ejection fraction (RVEF) were measured by cine MRI. The PR fraction (PRF) and MPA area were measured by 2D phase-contrast MRI. Spearman ρ values were determined to assess the relationships between circulation, RV function, and PRF. Vortex formation in the MPA occurred in 15 of 20 patients (75%). The longitudinal circulation (11.7 ± 5.1 m^2^/s) was correlated with the RVEF (ρ = − 0.85, p = 0.0002), RVEDVi (ρ = 0.62, p = 0.03), and RVESVi (ρ = 0.76, p = 0.003) after adjusting for the MPA size. The short-axis circulation (9.4 ± 3.4 m^2^/s) in the proximal MPA was positively correlated with the MPA area (ρ = 0.61, p = 0.004). The relationships between the PRF and circulation or RV function were not significant. Increased longitudinal circulation in the MPA, as demonstrated by circulation analysis using 4D flow MRI, was related to RV dysfunction in patients with a repaired TOF.

## Introduction

Pulmonary regurgitation (PR) is the most common complication after repaired Tetralogy of Fallot (TOF). It induces right ventricular (RV) volume overload, which leads to RV dilation and eventually RV dysfunction^1,2^. PR severity is reflected by the pulmonary regurgitant fraction [PRF (%)] measured on two-dimensional phase-contrast magnetic resonance imaging (2D PC-MRI), and is an important parameter for identifying when to replace the pulmonary valve^1^. However, there is a discrepancy between the PRF value, which can be underestimated, and symptom severity or RV function. One reason for an underestimation of the PRF is the presence of backward flow within a systole that is affected by turbulence or stagnation due to the dilation or stenosis of the pulmonary artery (PA) in patients after repair of the TOF^3^.

Four-dimensional (4D) flow MRI has been shown to be valuable for the qualitative and quantitative evaluation of pulmonary hemodynamics in patients with TOF^4–6^. Although flow patterns of the main pulmonary artery (MPA) in patients with a repaired TOF are characterized by increased helical or vortical flow features^4,5^, there are many variations because of PA dilation and stenosis. Therefore, an objective vortex flow analysis is needed. A vorticity analysis that quantifies intracardiac flow in patients with TOF and PA flow in patients with pulmonary hypertension has recently been reported to provide a new hemodynamic index^7–10^. Circulation is calculated from area integral of vorticity, and vorticity is defined as a spatial velocity derivative of flow velocity, and is affected by various factors, including pressure gradient, flow cohesiveness, and anatomic geometry^8,9^. Vorticity and circulation are the key measurements of rotation. Vorticity is the curl of the velocity field and provides information on the microscopic rotation, while circulation is a scalar integral quantity that provides information on the macroscopic rotation. We believe that the PRF alone does not reflect the true RV overload in patients with a repaired TOF, and that circulation analysis is a means by which to overcome its limitations. The purpose of this study was to investigate circulation analysis in the MPA by 4D flow MRI for assessment of MPA in patients after repair of the TOF by evaluating the relationship of vortex formation with RV function and PRF.

## Methods

### Participants

**Figure 1:**
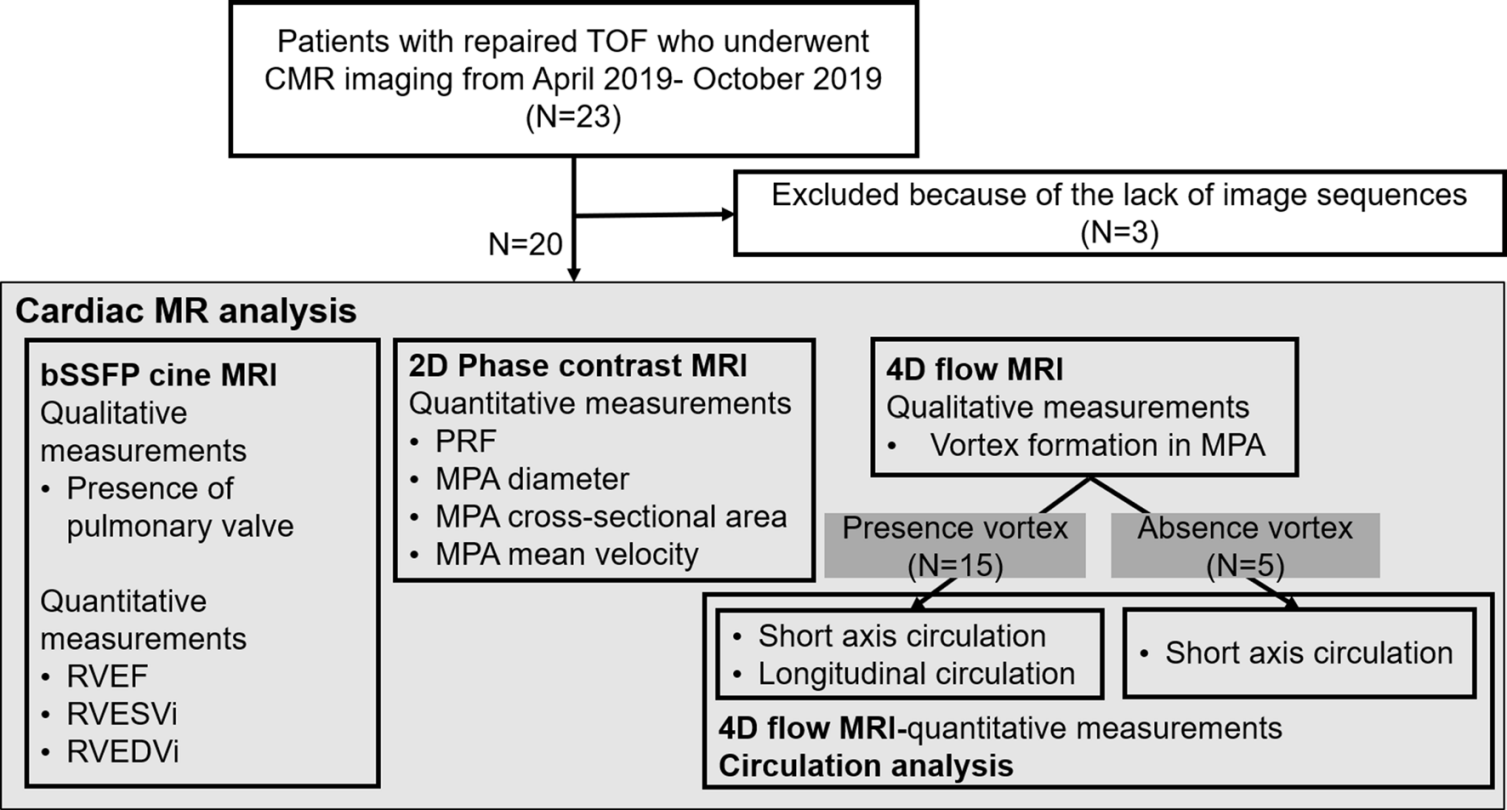
Study design and enrollment flow chart. *TOF* Tetralogy of Fallot, *CMR* cardiac magnetic resonance, *bSSFP* balanced steady-state free precession, *RV* right ventricular, *EF* ejection fraction, *EDVi* end-diastolic volume indexed for body surface area, *ESVi* end-systolic volume indexed for body surface area, *PRF* pulmonary regurgitant fraction, *MPA* main pulmonary artery.

This prospective study was approved by the institutional review board. Written informed consent was obtained from all the participating patients. For patients aged younger than 20 years, written informed consent was obtained from both the parents and the participating patient. The flowchart of the study is shown in Fig. 1.

The study initially enrolled 23 consecutive patients with repaired TOF from April 2019 to October 2019, who underwent cardiac magnetic resonance imaging (CMR). We excluded 3 patients who did not have the reconstructed 4D flow images required for circulation analysis. Finally, a total of 20 patients (10 men, 10 women with mean ages of 32 ± 13 years and 37 ± 13 years, respectively), who had CMR images of adequate quality for assessment were analyzed. Data on surgical procedures, mean pulmonary artery pressure (mPAP) as measured by right heart catheterization, and RV systolic pressure (RVSP) as measured by echocardiography were recorded from the electronic medical records. Information on right heart catheterization and echocardiography collected within 6 months from the MR examination was defined as valid data.

### CMR protocol

CMR imaging was part of routine clinical procedures and was performed with or without contrast media by a 3.0 T MR system (Ingenia 3 T; Phillips Healthcare, Best, the Netherlands) equipped with dual-source, parallel radiofrequency transmission, and a 32-element cardiac phased coil array for radiofrequency reception. The CMR clinical protocol included the following items: 2D multislice-cine-balanced-steady-state free precession (bSSFP) sequencing to quantify left ventricular (LV) and RV size and function; standard 2D-phase contrast sequencing to quantify PRF; and 4D-flow MRI. In 6 patients, intravenous (IV) administration of 0.1 mmol/kg gadoteric meglumine (Magnescope; Guerbet, Tokyo, Japan) was administered for late gadolinium-enhanced MRI. A cine-MR sequence was used to assess the end-systolic and end-diastolic volumes (ESV, EDV) of the ventricles, using the short-axis view and an axis view for the left and right ventricles, respectively, with the following settings: echo time/repetition time (TE/TR) 2 ms/3 ms, flip angle 50°, matrix size 192 × 141, and slice thickness 10 mm. ESV and EDV were normalized according to body surface area (BSA) and expressed as indices (EDVi, ESVi).The ejection fraction (EF) was defined as follows: EF (%) = ([EDV − ESV]/EDV) × 100. 2D-phase-contrast flow measurement was performed in the MPA with the following settings: TE/TR 2 ms/4 ms, flip angle 10°, matrix size 224 × 224, velocity encoding (VENC) 150–200 cm/s, 60 frames/cycle, and retrospective electrocardiogram (ECG) gating. PRF was defined as follows: PRF (%) = (reverse flow/forward flow) × 100. The mean velocity, maximum cross-sectional area, and maximum diameter of the proximal MPA were measured. A flow-sensitive 3D-gradient sequence with echo-planar imaging was used for 4D-flow MRI, with the following settings: TE/TR 3 ms/6 ms, flip angle 12°, matrix size 224 × 224, VENC 150–200 cm/s, 10 frame/cycle, and retrospective ECG gating. The presence of a visible pulmonary valve on cine MR was visually evaluated by 2 radiologists, who arrived at a consensus.

### 4D-flow MRI analysis

**Figure 2:**
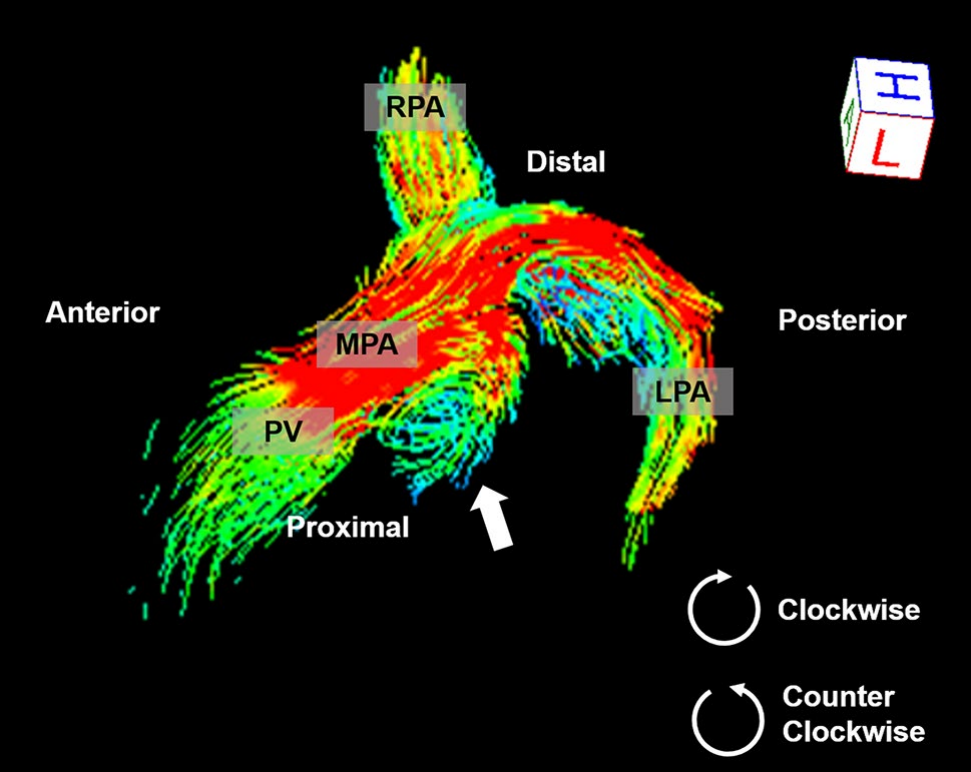
A 14-year-old boy with repaired Tetralogy of Fallot. Streamline imaging by 4D flow magnetic resonance imaging visualizes the pulmonary arteries. Clockwise vortices are seen at the posterior portion of the proximal main pulmonary artery when viewed from the patient’s left (arrow). Flow visualization was performed using 4D flow postprocessor software (iT Flow; Cardioflow Design. Inc., Tokyo, Japan). *MPA* main pulmonary artery, *LPA* left pulmonary artery, *RPA* right pulmonary artery.

Flow visualization of the MPA and quantification of circulation, which is an area integral of vorticity, were performed using 4D flow postprocessor software (iT Flow; Cardioflow Design. Inc., Tokyo, Japan)^11,12^. The magnitude of 4D-flow MRI and 4D-flow MR phase-specific images was used for anatomical segmentation of the pulmonary artery. The lumen of the MPA was segmented from the pulmonic valve to at least the first bifurcation into the right and left PAs. Pulmonary blood flow was visualized with the use of time-resolved 3D streamlines in the segmented luminal region of interest (ROI). The ROI was marked semiautomatically and corrected manually by the observer (N.T.). Vortex formation in the MPA throughout a cardiac cycle was qualitatively evaluated by 2 cardiothoracic radiologists (N.T. and M.N with greater than 10 years of experience each), who reached a consensus. Vortex formation was evaluated with regard to location (proximal or distal, anterior or posterior) and direction (clockwise or counterclockwise) (Fig. 2). The direction was assessed from the patient’s left side, with the left pulmonary artery anterior and the right pulmonary artery posterior, or from the view above the pulmonary artery.

**Figure 3:**
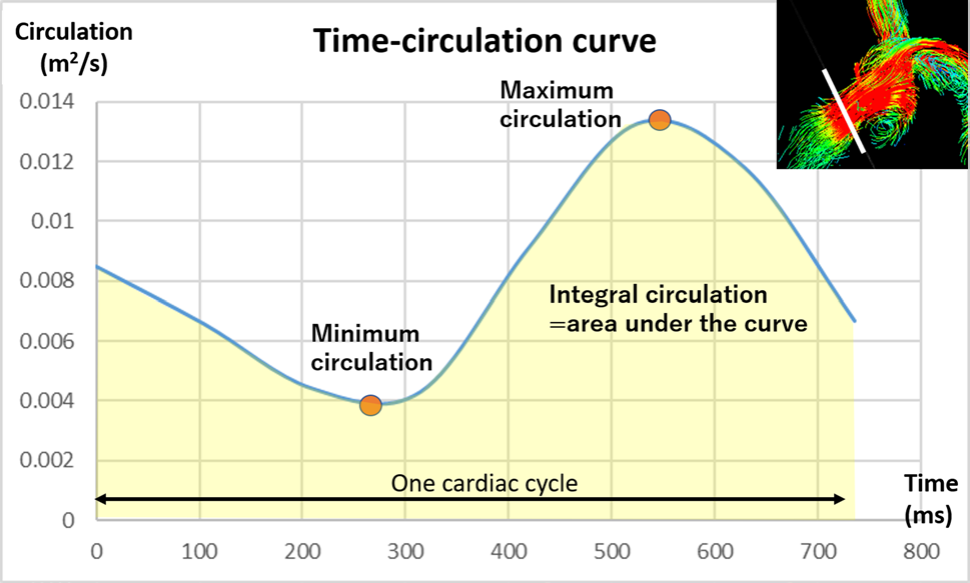
Time-circulation curve of the cross section of the proximal main pulmonary artery (white line in the right upper figure). The maximum and minimum values were obtained from the time- circulation curve. The integral value (area under the time-circulation curve = yellow area) was obtained by integration of the circulation values throughout a single cardiac cycle.

“Circulation” is defined in the following section. Maximum (Max), minimum (Min), and integral circulation were derived from a time-circulation curve (Fig. 3). Integral values were obtained by integration of the circulation measurements throughout a cardiac cycle.

**Figure 4:**
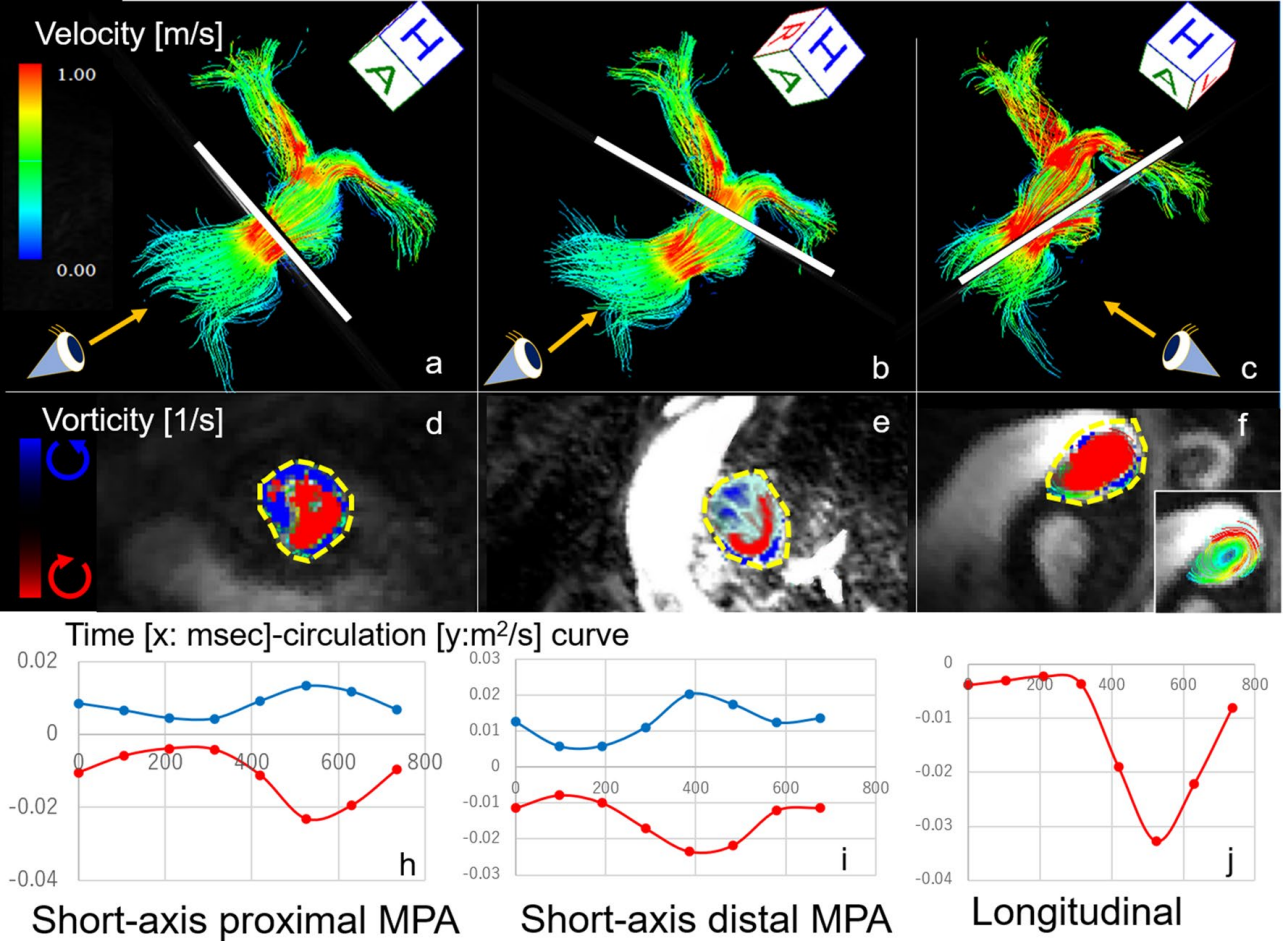
Streamline imaging flows of the pulmonary arteries of a 40-year-old man with repaired TOF (Case 6). The leftmost image and middle image show short-axis vorticities; the rightmost image shows a long-axis vorticity. The white lines in the uppermost row, where the vorticity was calculated, correspond to the cross sections shown in the middle row. The location of the measurement in the proximal MPA occurred at 1 cm above the pulmonic valve (**a**: white line). The location of the measurement in the distal MPA occurred at 1 cm below the bifurcation of the MPA into the right and left pulmonary arteries (**b**: white line). The longitudinal circulation was measured parallel to the clockwise systolic vortex in the MPA (**c**: white line). Vortex formation in the 3 different cross sections of the main pulmonary artery demonstrates the clockwise vortices in red and the counterclockwise vortices in blue [**d**: cross sections of the proximal MPA, **e**: cross section of the distal MPA, **f**: cross section of the vortex (middle row)]. The area surrounded by the yellow dotted line was the region where circulation (area integral of vorticity) was calculated (**d**: short-axis proximal MPA, **e**: short-axis distal MPA, **f**: longitudinal). The region of interest for the longitudinal circulation manually traced the vortex that can be recognized by streamline analysis (**f**: small square). The leftmost image and middle image show short-axis vorticities; the rightmost image shows a long-axis vorticity. The time-circulation curves show waveforms with 1 or 2 peaks (**h**: short-axis proximal MPA, **i**: short-axis distal MPA, **j**: longitudinal). The red line represents clockwise circulation and the blue line represents counterclockwise circulation (lowermost row). Flow visualization was performed with the use of 4D-flow postprocessor software (iT Flow; Cardioflow Design. Inc., Tokyo, Japan). Time-circulation curves were created by Microsoft Office Excel Ver 2101.

Short-axis circulation measurements were determined at specific cross-sectional regions of the proximal (Fig. 4a,d,h) and distal MPA (Fig. 4b,e,i) in all patients. Measurement of the short-axis circulation was established because that could be used to quantify circulation and consistently provide high reproducibility, whether or not the vortices could be detected visually. The measurement location in the proximal MPA occurred at 1 cm above the pulmonic valve (Fig. 4a). The measurement location in the distal MPA occurred at 1 cm below the MPA bifurcation into the right and left pulmonary artery (Fig. 4b). To assess short-axis circulation, clockwise and counterclockwise circulation were measured when viewed from the feet of the patient, with the pulmonary valve located anteriorly and the bifurcation of the pulmonary artery located posteriorly.

Long-axis cross-sectional circulation (longitudinal circulation) was measured in patients showing vortex formation in the MPA (Fig. 4c,f,j). Assessment of the longitudinal circulation allows the quantification of the strength of visually obvious vortices. The longitudinal circulation was measured parallel to the clockwise systolic vortex in the MPA (Fig. 4c). ROI manually traced the area of vortices that can be recognized by streamline analysis (Fig. 4f).

### Vorticity and circulation formula

The definition of vorticity is as follows:

1 $$ Vorticity = curl\;(\vec{U}) = \left( {\frac{{\partial w}}{{\partial y}} - \frac{{\partial v}}{{\partial z}},\;\frac{{\partial u}}{{\partial z}} - \frac{{\partial w}}{{\partial x}},\;\frac{{\partial v}}{{\partial x}} - \frac{{\partial u}}{{\partial y}}} \right) $$

In Eq. (1), x: x direction, y: y direction, z: z direction, u: velocity in x direction, v: velocity in y direction, w: velocity in z direction.

Definition of 2D vorticity is as follows:

2 $$ 2D \;vorticity = \frac{\partial v}{{\partial x}} - \frac{\partial u}{{\partial y}} $$

3 $$ Circulation = \smallint vorticity \cdot dS $$

In Eqs. (2), (3), x: differential in x direction, y: differential in y direction, u: velocity in x direction at a cross-section, v: velocity in y direction at a cross-section, S: pixel area.

The software (iTflow) calculation is for 2D vorticity was as follows:

4 $$ 2D \;vorticity = \frac{\partial v}{{\partial x}} - \frac{\partial u}{{\partial y}} = \frac{v(i + 1, \;j) - v(i - 1,\;j)}{{2*pixel\;pitch\; x}} - \frac{u(i,\;j + 1) - u(i,\;j - 1)}{{2*pixel \;pitch \;y}} $$

5 $$ Circulation = \sum 2D \;vorticity*pixel \;pitch\; X*pixel \;pitch\; Y $$

6 $$ Clockwise\;circulation = \mathop{\sum}\limits_{2D \;vorticity > 0} 2D \;vorticity*pixel\; pitch\; X*pixel \;pitch\; Y $$

7 $$ Counter\;clockwise\;circulation = \left| {\mathop \sum \limits_{2D\; vorticity < 0} 2D \;vorticity*pixel \;pitch \;X*pixel \;pitch\; Y} \right| $$

In Eq. (4), ∂x: differential in the x direction, ∂y: differential in the y direction, u: velocity in the x direction at a cross-section, v: velocity in the y direction at a cross-section, i: pixel index in the x direction at a cross-section, and, j: pixel index in the y direction at a cross-section. The spatial gradient velocity was calculated by the central difference.

### Statistical analysis

JMP 11 (SAS Institute Japan, Tokyo, Japan) was used to perform statistical analysis. Continuous variables are expressed as medians plus interquartile range. Categorical variables are expressed as numerals and percentages. The Spearman correlation coefficient was determined to assess the correlation between circulation and other measurements (RVEF, RVEDVi, EVESVi, PRF, MPA maximum area, MPA maximum diameter, MPA velocity, Age, BSA, mPAP, RVSP). Partial correlation analysis was performed by MPA measurement into a control variable to determine the relationship between the circulation and RV function as multivariant analysis. Three different multivariate model were applied to assess the role of MPA measurement (control variable: 1. MPA maximum area; 2. MPA maximum diameter; 3. MPA maximum area and diameter) in the relation between circulation and RV function. Receiver operating characteristics (ROC) curve were constructed to assess the ability of circulation and PRF to predict RVEF less than 50%. The sensitivity, specificity, and area under the curve (AUC) were calculated. A p-value less than 0.05 was considered statistically significant.

### Ethical approval

All procedures performed in studies involving human participants were in accordance with the ethical standards of the Ethics Committee for Clinical Research of Tokyo Women's Medical University (approval number, 180309) and with the 1964 Helsinki declaration and its later amendments or comparable ethical standards.

### Consent to participate

Informed consent was obtained from all the individual participants included in the study.

## Results

### Participants

Patient characteristics and basic CMR imaging results are summarized in Table 1 (detailed information in Supplemental Table S1). A patch infundibuloplasty was performed in 9 of 20 patients (45%); transannular patch repair was performed in 6 patients (30%); pulmonary valve replacement was performed in 2 patients (10%); and conotruncal repair, muscle resection, and Rastelli procedure were each performed in 1 patient (5% each). The pulmonary valve could be confirmed visually on cine MRI in 11 patients.

**Table 1:** Characteristics of 20 patients with repaired Tetralogy of Fallot.

	N = 20
Age (years)	39 (range 12–59)
Sex (male, female)	10, 10
BSA (m^2^)	1.55 (1.4, 1.7)
Heart rate (bpm)	71 (63, 76)
LV EF (%)	45 (38, 52)
LV EDVi (mL/m^2^)	75 (64, 85)
LV ESVi (mL/m^2^)	39 (34, 49)
RV EF (%)	55 (49, 61)
RV EDVi (mL/m^2^)	129 (110, 175)
RV ESVi (mL/m^2^)	62 (41, 76)
MPA cross-sectional area (cm^2^)	8.5 (6.6, 9.5)
MPA diameter (cm)	32 (28, 35)
MPA mean velocity (cm/s)	11.2 (10.3, 16.2)
Pulmonary regurgitant fraction (%)	41 (35, 45)
Mean pulmonary artery pressure (mmHg) n = 14	13.5 (12, 18)
Right ventricular systolic pressure (mmHg) n = 16	41 (39, 46)

Continuous variables were expressed as median and interquartile range (lower, upper).

*BSA* body surface area, *LV* left ventricular, *EF* ejection fraction, *EDVi* end-diastolic volume indexed for body surface area, *ESVi* end-systolic volume indexed for body surface area, *RV* right ventricular, *MPA* main pulmonary artery.

### Flow visualization

**Figure 5:**
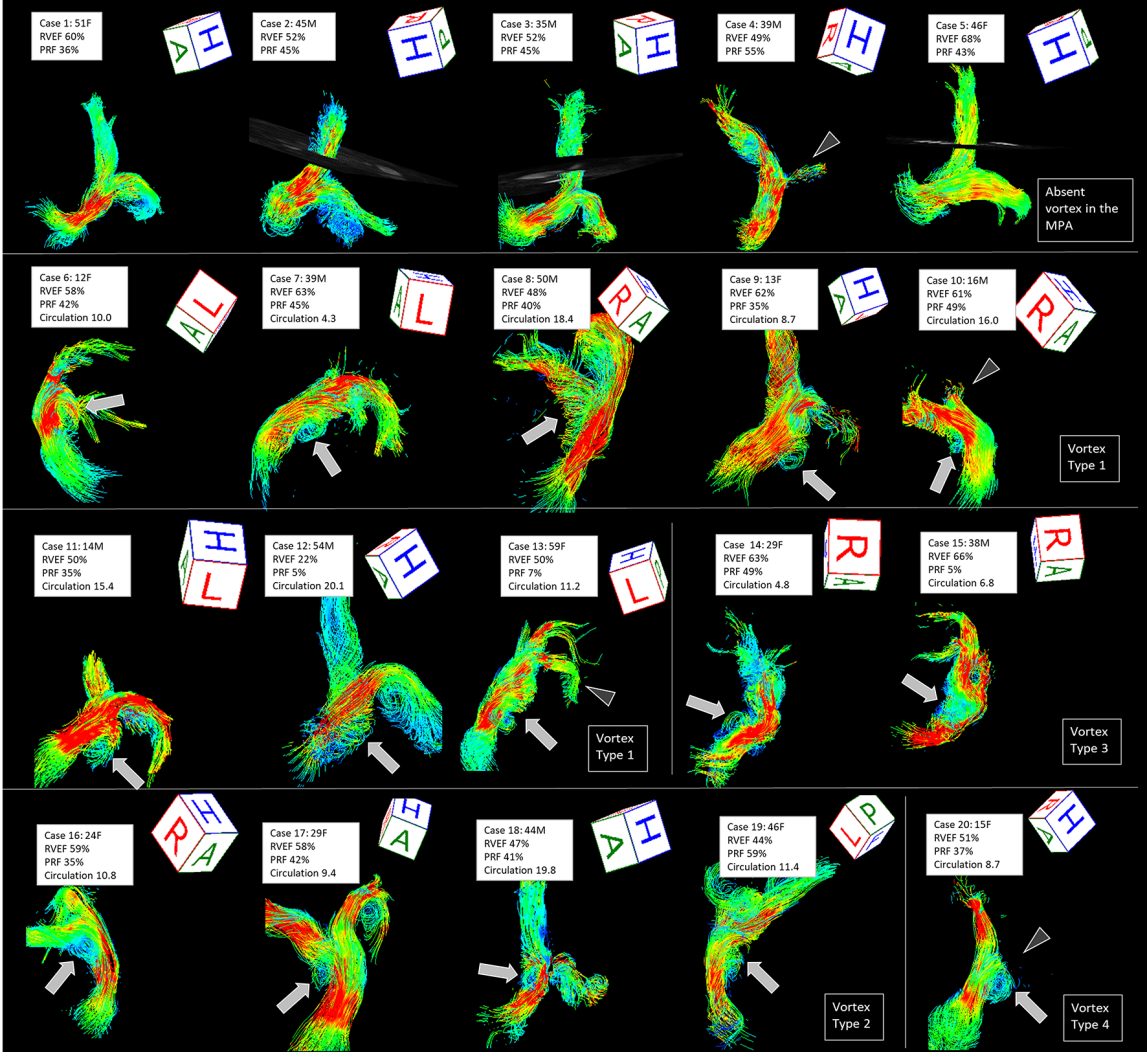
Streamline imaging flows of the pulmonary arteries of 20 patients with repaired Tetralogy of Fallot. Case 1–5 did not show a vortex in the main pulmonary arteries (MPA), however, there were vortical or helical flows in the right and left PA in some patients. Case 6–20 showed vortex formation in the MPA. Vortices typically originated posteriorly in the MPA (Type 1: Case 6–13) and formed helices. Other vortices originated from the MPA bifurcation to the right PA (Type 2: Case 16–19), at the proximal right PA (Type 3: Case 14, 15), or from the MPA bifurcation to the left PA (Type 4: Case 20). Arrows indicate vortices and arrowheads indicate the left pulmonary artery stenosis. *F* female, *M* male, *RVEF* right ventricular ejection fraction, *PRF* pulmonary regurgitant fraction, *Circulation* longitudinal integral circulation value. Flow visualization was performed using 4D flow postprocessor software (iT Flow; Cardioflow Design. Inc., Tokyo, Japan).

Four-dimensional flow from the MPA to the RPA was visualized in all 20 patients (Fig. 5). LPA flow could not be visualized in 4 patients. One of these patients showed artifacts due to an implanted stent in the left PA (Case 20), and 3 patients showed marked stenosis of the LPA (Case 4, 10, 13). Four-dimensional flow imaging showed variations in the outflow from the RV to the MPA in patients repaired by various surgical procedures. None of the patients had pulmonary atresia. Vortices in the MPA were seen in 15 of 20 (75%) patients. Most of the vortices appeared larger in systole than in diastole. Vortices typically originated posteriorly in the MPA (Type 1: 53%, 8/15) and formed helices. Other vortices originated from the MPA bifurcation to the RPA (Type 2: 26%, 4/15), at the proximal RPA (Type 3: 13%, 2/15), or from the MPA bifurcation to the LPA (Type 4: 6%, 1/15). Viewed from the right side of the patient, the vortices appeared clockwise (100%, 15/15) in the dilated MPA (Figs. 2 and 4). No vortices were seen in the MPA of 5 (25%) patients. In patients without a vortex, the shape of the MPA was almost normal, and neither eccentric enlargements nor barrel-like deformations were seen.

### Correlation between vorticity and CMR imaging parameters

The results of circulation analysis in the MPA are summarized in Table 2. The Max and Integral values for clockwise short-axis vorticity were greater than those for counterclockwise circulation. The time-circulation curves of short-axis circulation in the MPA showed waveforms with 1 or 2 peaks, and the waves of the clockwise circulation and counterclockwise circulation were almost parallel. The time-circulation curve of longitudinal circulation showed a single waveform with 1 peak (Fig. 4).

**Table 2:** Circulation in the main pulmonary artery was derived from 4D flow MRI. The Max and Integral values for clockwise short-axis vorticity were greater than those for counterclockwise circulation.

	Clockwise	Counter clockwise
**Short-axis circulation proximal, N = 20**		
Max (m^2^/s)	0.018 (0.013, 0.024)	0.017 (0.013, 0.021)
Min (m^2^/s)	0.004 (0.002, 0.006)	0.004 (0.003, 0.006)
Integral (m^2^)	9.1 (6.4, 11.3)	7.7 (6.7, 10.7)
**Short-axis circulation distal, N = 20**		
Max (m^2^/s)	0.024 (0.017, 0.033)	0.017 (0.015, 0.025)
Min (m^2^/s)	0.005 (0.004, 0.008)	0.005 (0.004, 0.007)
Integral (m^2^)	10.1 (8.1, 14.1)	8.6 (7.2, 12.1)
**Longitudinal circulation, N = 15**		
Max (m^2^/s)	0.032 (0.015, 0.049)	–
Min (m^2^/s)	0.003 (0.002, 0.006)	–
Integral (m^2^)	10.7 (8.7, 15.9)	–

Continuous variables are expressed as medians plus interquartile range (lower, upper).

Clockwise circulation in the short-axis proximal MPA had significant correlation with RVEDVi (Max ρ = 0.46, p = 0.03; Integral ρ = 0.47, p = 0.04) and RVESVi (Max ρ = 0.49, p = 0.02; Integral ρ = 0.49, p = 0.03; Table 3). The area of the MPA was positively correlated with clockwise (Max ρ = 0.54, p = 0.01; Integral ρ = 0.59, p = 0.006) and counterclockwise circulation in the proximal MPA (Max ρ = 0.66, p = 0.001; Integral ρ = 0.61, p = 0.004; Table 4). The diameter of the MPA was positively correlated with counterclockwise circulation in the proximal MPA (Max ρ = 0.50, p = 0.02; Min ρ = 0.46, p = 0.03; Table 4). Based on partial correlation analysis, after adjustment for the MPA area, the correlation between the short-axis proximal MPA and RV measurement was not significant (Table 5).

**Table 3:** Correlation between circulation in the main pulmonary artery and right ventricular function. Clockwise circulation in the short-axis proximal MPA had significant correlation with RVEDVi. Counterclockwise circulation in the short-axis distal MPA had a significant negative correlation with RVEF. The clockwise longitudinal circulation had significant correlations with the RVEF, RVEDVi, and RVESVi.

Circulation	RV EF		RV EDVi		RV ESVi		Age		BSA	
	ρ	P	ρ	P	ρ	P	ρ	P	ρ	P
**Short-axis proximal (N = 20)**										
Clockwise										
Max	− 0.18	0.4	**0.46**	**0.03**	**0.49**	**0.02**	0.31	0.1	0.09	0.6
Min	− 0.34	0.1	0.19	0.4	− 0.27	0.2	0.4	0.07	0.28	0.2
Integral	− 0.21	0.3	**0.47**	**0.04**	**0.49**	**0.03**	0.28	0.2	0.25	0.2
Counter clockwise										
Max	− 0.16	0.5	0.28	0.2	0.3	0.1	0.4	0.07	0.18	0.4
Min	− 0.42	0.06	0.1	0.6	− 0.03	0.8	0.29	0.2	0.21	0.3
Integral	− 0.17	0.4	0.31	0.1	0.3	0.1	0.26	0.2	0.3	0.1
**Short-axis distal (N = 20)**										
Clockwise										
Max	− 0.24	0.3	0.24	0.2	0.32	0.1	0.07	0.7	0.01	0.9
Min	− 0.3	0.1	0.2	0.3	0.25	0.2	0.11	0.6	0.09	0.7
Integral	− 0.19	0.4	0.25	0.2	0.29	0.2	0.07	0.7	0.02	0.9
Counter clockwise										
Max	− 0.3	0.1	0.001	0.9	0.13	0.5	0.2	0.3	0.19	0.3
Min	− **0.52**	**0.01**	0.06	0.7	0.22	0.3	0.14	0.5	0.02	0.9
Integral	− 0.23	0.3	0.11	0.6	0.18	0.4	0.11	0.6	0.1	0.6
**Longitudinal (N = 15)**										
Clockwise										
Max	− **0.76**	**0.0009**	0.43	0.1	**0.62**	**0.01**	0.32	0.2	0.07	0.7
Min	− **0.65**	**0.009**	− 0.01	0.9	0.23	0.4	0.48	0.06	0.26	0.3
Integral	− **0.8**	**0.0003**	**0.56**	**0.02**	**0.73**	**0.002**	0.35	0.1	0.08	0.7

p-value (P) from Spearman signed rank test. Boldface indicates statistically significance (p < 0.05).

*RV* right ventricular, *EF* ejection fraction, *EDVi* end-diastolic volume indexed for body surface area, *ESVi* end-systolic volume indexed for body surface area, *BSA* body surface area.

**Table 4:** Correlation between pulmonary artery measurements and circulation or right ventricular function. The area of the MPA was positively correlated with clockwise and counterclockwise circulation in the proximal MPA. The diameter of the MPA was positively correlated with counterclockwise circulation in the proximal MPA. The diameter of the MPA was positively correlated with clockwise and counterclockwise Min circulation in the short-axis distal MPA. The diameter of the MPA was positively correlated with the Min longitudinal circulation.

Circulation	MPA-Area-max		MPA diameter max		PRF		mPAP		RVSP		MPA velocity	
	ρ	P	ρ	P	ρ	P	ρ	P	ρ	P	ρ	P
N	20		20		20		14		16		20	
**Short-axis proximal MPA**												
CW												
Max	**0.54**	**0.01**	0.34	0.1	− 0.006	0.9	− 0.48	0.08	− 0.12	0.6	− 0.17	0.4
Min	0.34	0.1	0.37	0.1	− 0.16	0.4	− 0.52	0.05	− 0.07	0.7	− 0.32	0.1
Integral	**0.59**	**0.006**	0.29	0.2	− 0.13	0.5	− 0.41	0.1	− 0.06	0.7	− 0.16	0.4
CCW												
Max	**0.66**	**0.001**	**0.5**	**0.02**	0.03	0.8	− 0.28	0.3	0.03	0.8	− 0.26	0.2
Min	0.34	0.1	**0.46**	**0.03**	− 0.06	0.7	− 0.15	0.5	0.14	0.5	− 0.38	0.09
Integral	**0.61**	**0.004**	0.36	0.1	0.03	0.8	− 0.1	0.7	0.12	0.6	− 0.09	0.7
**Short-axis distal MPA**												
CW												
Max	0.33	0.1	0.2	0.3	− 0.27	0.2	− 0.09	0.7	0.43	0.07	0.08	0.7
Min	0.34	0.1	**0.73**	**0.0002**	− 0.03	0.8	− 0.14	0.6	0.17	0.4	− 0.26	0.2
Integral	0.33	0.1	0.25	0.2	− 0.17	0.4	0.02	0.9	**0.48**	**0.04**	0.22	0.3
CCW												
Max	0.1	0.6	0.2	0.3	− 0.07	0.7	− 0.02	0.9	0.4	0.09	0.17	0.4
Min	0.28	0.2	**0.65**	**0.001**	− 0.36	0.1	− 0.16	0.5	0.22	0.3	− 0.29	0.2
Integral	0.24	0.3	0.2	0.3	0.03	0.8	− 0.15	0.5	0.33	0.2	0.29	0.2
N	15		15		15		11		14		15	
**Longitudinal**												
CW												
Max	0.1	0.6	0.11	0.6	− 0.17	0.5	− 0.2	0.5	0.04	0.8	− 0.03	0.8
Min	0.1	0.6	**0.54**	**0.03**	− 0.32	0.2	0.19	0.5	0.24	0.3	− 0.45	0.09
Integral	0.1	0.6	0.05	0.8	− 0.14	0.6	− 0.1	0.7	0.13	0.6	− 0.007	0.9
N	20		20		20		14		16		20	
**Right ventricular function**												
RVEF	0.16	0.4	− 0.55	0.8	0.03	0.8	− 0.02	0.9	− 0.39	0.1	0.04	0.8
RVEDVi	**0.45**	**0.04**	0.07	0.7	− 0.09	0.7	− 0.05	0.8	− 0.04	0.8	− 0.03	0.8
EVESVi	0.24	0.3	0.03	0.8	− 0.1	0.6	− 0.11	0.6	0.15	0.5	− 0.006	0.9
PRF	− 0.14	0.5	− 0.18	0.4	–	–	0.04	0.8	− 0.05	0.8	0.36	0.1

P-value (P) from Spearman signed rank test. Boldface indicates statistical significance (p < 0.05).

*MPA* main pulmonary artery, *PRF* pulmonary regurgitant fraction, *mPAP* mean pulmonary artery pressure, *RVSP* right ventricular systolic pressure, *RV* right ventricular, *EF* ejection fraction, *EDVi* end-diastolic volume indexed for body surface area, *ESVi* end-systolic volume indexed for body surface area, *CW* clockwise, *CCW* counter clockwise.

**Table 5:** The partial correlation coefficient between the circulation and right ventricular function adjusted based on the size of the pulmonary artery. After adjustment of the area of the MPA, the partial correlation between the short-axis proximal MPA and RV measurement was not significant. Partial correlation analysis after adjustment based on the diameter of the MPA showed a significant correlation between the counterclockwise Min circulation in the short-axis distal MPA and RVEF. Partial correlation analysis, after adjustment based on the area of the MPA, showed a significant correlation between the clockwise longitudinal circulation and RVEF, RVEDVi, and RVESVi.

Adjusted parameter	MPA-Area max						MPA diameter max						MPA-Area max + MPA diameter max					
Circulation	RV EF		RV EDVi		RV ESVi		RV EF		RV EDVi		RV ESVi		RV EF		RV EDVi		RV ESVi	
	ρ	P	ρ	P	ρ	P	ρ	P	ρ	P	ρ	P	ρ	P	ρ	P	ρ	P
**Short-axis proximal**																		
CW																		
Max	− 0.33	0.17	*0.30*	*0.22*	*0.44*	*0.06*	− 0.17	0.48	***0.47***	***0.04***	***0.51***	***0.02***	− 0.33	0.17	*0.31*	*0.21*	*0.44*	*0.07*
Min	− 0.34	0.06	0.05	0.85	0.28	0.25	− 0.35	0.14	0.18	0.46	0.35	0.14	− 0.41	0.09	0.11	0.66	0.32	0.19
Integral	− 0.39	0.10	*0.28*	*0.24*	*0.44*	*0.06*	− 0.21	0.39	***0.47***	***0.04***	***0.51***	***0.03***	− 0.43	0.07	*0.26*	*0.30*	*0.43*	*0.08*
CCW																		
Max	− 0.38	0.11	− 0.02	0.93	0.19	0.43	− 0.17	0.48	0.29	0.23	0.34	0.16	− 0.36	0.14	0.03	0.92	0.22	0.37
Min	− **0.53**	**0.02**	− 0.06	0.80	0.18	0.45	− 0.46	0.05	0.08	0.76	0.27	0.26	− 0.50	0.04	0.05	0.84	0.26	0.29
Integral	− 0.36	0.13	0.05	0.84	0.20	0.42	− 0.18	0.46	0.30	0.21	0.31	0.19	− 0.38	0.12	0.04	0.88	0.19	0.45
**Short-axis distal**																		
CW																		
Max	− 0.32	0.18	0.09	0.72	0.25	0.31	− 0.24	0.33	0.21	0.38	0.31	0.20	− 0.33	0.18	0.09	0.72	0.25	0.32
Min	− 0.38	0.11	0.06	0.81	0.22	0.36	− 0.39	0.10	0.22	0.37	0.39	0.10	− 0.35	0.16	0.42	0.08	**0.50**	**0.03**
Integral	− 0.26	0.28	0.13	0.61	0.23	0.35	− 0.18	0.45	0.24	0.31	0.29	0.23	− 0.26	0.30	0.15	0.55	0.24	0.34
CCW																		
Max	− 0.32	0.18	− 0.05	0.84	0.11	0.65	− 0.30	0.22	− 0.01	0.95	0.13	0.60	− 0.30	0.23	0.01	0.98	0.15	0.56
Min	− **0.60**	**0.01**	− 0.08	0.75	0.21	0.39	− **0.65**	**0.002**	0.02	0.95	0.33	0.17	− **0.63**	**0.005**	0.17	0.51	0.43	0.08
Integral	− 0.28	0.24	0.01	0.97	0.14	0.57	− 0.23	0.35	0.10	0.67	0.19	0.44	− 0.28	0.26	0.03	0.91	0.15	0.54
**Longitudinal**																		
CW																		
Max	− ***0.80***	***0.001***	0.43	0.13	***0.62***	***0.02***	− ***0.77***	***0.001***	0.45	0.11	***0.65***	***0.01***	− ***0.80***	***0.001***	0.50	0.09	***0.66***	***0.01***
Min	− ***0.69***	***0.006***	− 0.06	0.83	0.21	0.47	− ***0.80***	***0.001***	0.04	0.89	0.36	0.21	− ***0.79***	***0.001***	0.24	0.44	0.46	0.11
Integral	− ***0.85***	***0.0001***	***0.57***	***0.03***	***0.73***	***0.00***	− ***0.81***	***0.001***	***0.57***	***0.03***	***0.75***	***0.002***	− ***0.85***	***0.0002***	***0.62***	***0.03***	***0.76***	***0.003***

P-value (P) from Spearman signed rank test. Boldface indicates statistical significance (p < 0.05). Italic typeface indicates statistical significance by univariate analysis (p < 0.05 in Table 4).

*MPA* main pulmonary artery, *RV* right ventricular, *EF* ejection fraction, *EDVi* end-diastolic volume indexed for body surface area, *ESVi* end-systolic volume indexed for body surface area, *CW* clockwise, *CCW* counter clockwise.

Counterclockwise circulation in the short-axis distal MPA had a significant negative correlation with RVEF (Min ρ = − 0.52; Table 3). The diameter of the MPA was positively correlated with clockwise and counterclockwise Min circulation in the short-axis distal MPA (clockwise ρ = 0.73, p = 0.0002; counterclockwise ρ = 0.65, p = 0.001; Table 4). Partial correlation analysis, after adjustment for the MPA diameter, had a significant correlation between the counterclockwise Min circulation in the short-axis distal MPA and RVEF (Min ρ = − 0.65, p = 0.002; Table 5).

**Figure 6:**
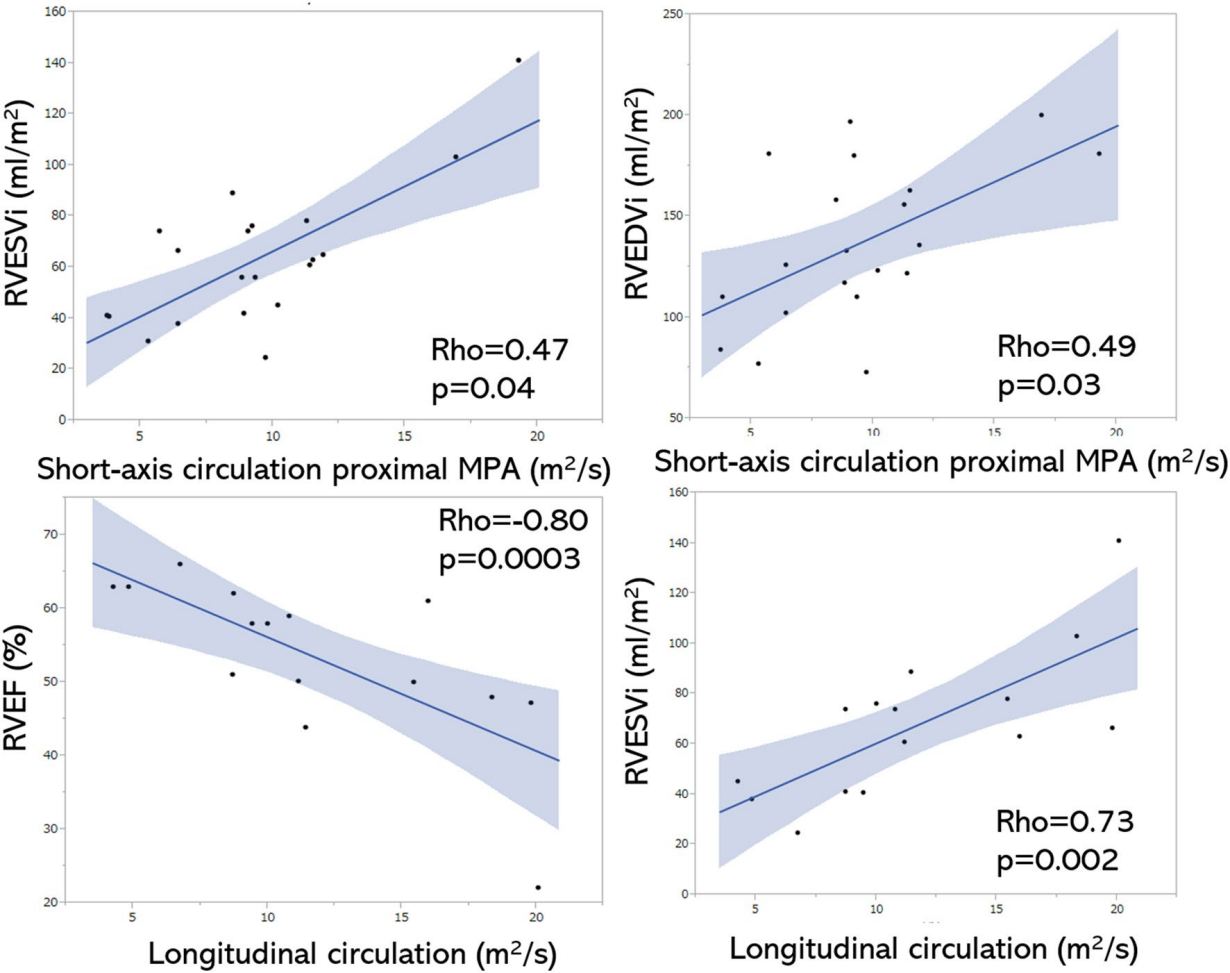
Correlations between integral circulation and RV functions. The short-axis integral circulations in the proximal MPA show significant positive correlations with RVESVi and RVEDVi (upper 2 graphs). The longitudinal integral circulation shows a negative correlation with RVEF and a positive correlation with RVESVi (lower 2 graphs).

The clockwise longitudinal circulation had significant correlations with the RVEF (Max ρ = − 0.76, p = 0.0009; Min ρ = − 0.65, p = 0.009; Integral ρ = − 0.80, p = 0.0003), RVEDVi (Integral ρ = 0.56, p = 0.02) and RVESVi (Max ρ = 0.62, p = 0.01; Integral ρ = 0.73, p = 0.002; Table 3, Fig. 6). The diameter of the MPA was positively correlated with the Min longitudinal circulation (ρ = 0.54, p = 0.03; Table 4). Partial correlation analysis, after adjustment for MPA area, showed a significant correlation between the clockwise longitudinal circulation and RVEF (Max ρ = − 0.80, p = 0.001; Min ρ = − 0.79, p = 0.001; Integral ρ = − 0.85, p = 0.0002), RVEDVi (Integral ρ = 0.62, p = 0.03) and RVESVi (Max ρ = 0.66, p = 0.01; Integral ρ = 0.76, p = 0.003; Table 5).

The correlations between the PRF and circulation, and between the PRF and RV function were not significant. The correlation between the mean velocity of the MPA and the circulation was not significant. The correlations between the mean the pulmonary artery pressure and circulation, and between the RV systolic pressure and circulation were not significant.

The sensitivity and specificity of the longitudinal circulation predicting RVEF less than 50% was as follow: Max longitudinal circulation sensitivity = 75%, specificity = 75%, AUC = 0.89, cutoff value = 0.051 m^2^/s; Min longitudinal circulation sensitivity = 75%, specificity = 57%, AUC = 0.82, cutoff value = 0.0059 m^2^/s; Integral longitudinal circulation sensitivity = 100%, specificity = 82%, AUC = 0.95, cutoff value = 11.4). The sensitivity and specificity of the PRF predicting RVEF less than 50% was 40% and 40% (AUC = 0.59, cutoff value = 55%), respectively.

## Discussion

This study demonstrated that the increased longitudinal circulation in the MPA, as demonstrated by 4D-flow MRI, was significantly correlated with a reduced RVEF and increased RVESVi after adjustment for MPA size in patients with a repaired TOF. In contrast, an increased circulation was not associated with the PRF.

In agreement with previous 4D flow MRI studies involving patients with a repaired TOF, our study revealed various patterns of vortex formation in the MPA. Francois et al.^5^ reported that more than half of their patients with repaired TOF had helical and vortical flow patterns in the MPA during the late systolic phase. Moreover, Geiger et al.^4^ concluded that a vortex in the MPA coincides with an increasing outflow tract size that encompasses the circular flow. Geiger et al.^4^ discussed the relationship between vortex formation and dilated pulmonary arteries, but could not determine which of the two factors preceded the other. In the current study, 25% of patients did not exhibit a vortex, and the PA morphology was normal. The short-axis circulation, which reflects inhomogeneity of flow in the MPA cross section, was positively correlated with the size of the MPA. The MPA area correlated with systolic circulation (maximum circulation) in proximal MPA, whereas the diameter of the MPA correlated with diastolic circulation (minimum circulation) in distal MPA. This discrepancy might be accounted for by the shape of the cross-section of the MPA, which is an ellipse, rather than a circle.

Reiter et al.^13–15^ reported that the duration of vortical flow in the MPA is correlated with elevation of the pulmonary artery pressure. The integral circulation increases with the increasing duration of vortical flow. We speculate that the development of vortex flow is associated with increased distal pulmonary vascular resistance, or pulmonary hypertension. In the current study the mean pulmonary artery pressure for the patient group was 14 mmHg, and no patients had pulmonary hypertension. The relationship between integral circulation and pulmonary artery pressure could not be confirmed.

In the current study, all patients had pulmonary regurgitation with PRF > 5%. Thus, the pulmonary artery flow had a to-and-fro pattern in the long axis direction throughout the cardiac cycle. We suggest that this to-and-fro pattern is largely responsible for the onset of the longitudinal vortex. The antegrade flow in early systole from the pulmonary valve to the left and right pulmonary arteries (beyond bifurcation) was stagnated at the end of systole. Then, due to pulmonary regurgitation, retrograde flow joins in the MPA at the onset of diastole. We assume that the to-and-fro pattern between the MPA and the bifurcation is one of the causes underlying vortex flow generation, and if there is more to-and-fro flow, the longitudinal vortex and circulation will become larger. Increased to-and-fro flow at the RV outlet is consistent with RV overload. There is a lack of reproducibility in the PRF by 2D-phase contrast MRI measured in just one transverse cross-section. If a vortex is present in the MPA, phase-contrast MRI may underestimate the volume because of intravoxel dephasing and a loss of signal^16^. The tendency to underestimate is enhanced for the volume of backward flow during diastole. Furthermore, it is difficult to accurately scan the orthogonal cross-section of the dilated and tortuous MPA. Our vorticity analysis is a method that has the potential to compensate for the limitations of conventional PFR measurements.

This study had several limitations. First, the number of patients was small, and the study population included patients with varying degrees of PR severity who had undergone various surgical procedures for TOF repair. The elasticity and wall shear stress of the RV outflow tract and MPA vary, depending on the repair technique and the material used for replacement. These factors are considered important in the formation of a vortex and increased circulation. There was an insufficient number of patients for each procedure, thus comparing vorticities produced by each surgical procedure was not possible. Second, the study lacked clinical information for some study patients, which included signs and symptoms, results of laboratory testing (e.g.. N-terminal fragment of pro-B-type natriuretic peptide), and right heart catheterization findings. Third, there are few officially accepted and standardized methods of assessment of vortex flow and circulation in live study subjects^17^. At present, the clinical applicability of the circulation is limited because analysis of the circulation is based on the quality of the MRI and the need for specialized analytical software. Standardization of vortex flow analysis remains a future goal. In addition, the limited number of phases (only 10 phases/cycle) can lead to errors in measurements; the ideal number is 30 phases/cycle, which is based on recommendations by a consensus statement^18^. Performing the ideal assessment, however, would be difficult given the limited time of routine clinical examinations. Helicity is another approach for quantifying the vortex; however, helicity is an index that depends on the 3D morphology of the pulmonary artery. For that measurement, it is necessary to determine the starting and ending points of the pulmonary artery. Since there are many individual differences in the morphology of the pulmonary artery in TOF, standardizing the range of measurements for helicity of the pulmonary artery was difficult. Therefore, we decided to measure circulation, which can be calculated from a single cross-section.

In conclusion, increased longitudinal circulation in the MPA, as measured by 4D-MR flow analysis, was shown to be independently associated with right ventricular dysfunction in patients with a repaired TOF. Indeed, the circulation can serve as a new quantitative index for the contrast-free evaluation of heart overload in valvular and congenital heart disease.

## Supplementary Information

Supplementary Table S1.

## Data Availability

The datasets generated during and/or analyzed during the current study are available from the corresponding author on reasonable request.
