# Supplementary material for: Circulation derived from 4D flow MRI correlates with right ventricular dysfunction in patients with tetralogy of Fallot
Source: Sci Rep. 2021 Jun 2;11:11623. doi: 10.1038/s41598-021-91125-2 (PMC8172849; doi:10.1038/s41598-021-91125-2)
Supplement: Supplementary file 1 — Supplementary Table S1. [file 41598_2021_91125_MOESM1_ESM.docx]

| Supplemental Table 1. Detail information | | | | | | | | | | | |
| --- | --- | --- | --- | --- | --- | --- | --- | --- | --- | --- | --- |
| Case | Age | sex | Vortex | Type | RVEF | RVEDVi | RVESVi | PRF | mPAP | RVSP | Operation |
| 1 | 51 | F | Absent | NA | 60 | 77 | 31 | 36 | NA | NA | Patch infundibuloplasty |
| 2 | 45 | M | Absent | NA | 52 | 136 | 65 | 45 | 12 | 40 | Transannular patch |
| 3 | 35 | M | Absent | NA | 52 | 117 | 56 | 45 | 13 | 39 | Patch infundibuloplasty |
| 4 | 39 | M | Absent | NA | 49 | 110 | 56 | 55 | NA | 46 | Rastelli |
| 5 | 46 | F | Absent | NA | 68 | 133 | 42 | 43 | 18 | 39 | PVR |
| 6 | 12 | F | Present | type 1 | 58 | 180 | 76 | 42 | NA | 45 | Conotruncal repair |
| 7 | 39 | M | Present | type 1 | 63 | 123 | 45 | 45 | 8 | 35 | Patch infundibuloplasty |
| 8 | 50 | M | Present | type 1 | 48 | 200 | 103 | 40 | 15 | 40 | Transannular patch |
| 9 | 13 | F | Present | type 1 | 62 | 197 | 74 | 35 | 24 | 51 | Transannular patch |
| 10 | 16 | M | Present | type 1 | 61 | 163 | 63 | 49 | 12 | 41 | Transannular patch |
| 11 | 14 | M | Present | type 1 | 50 | 156 | 78 | 35 | 12 | NA | Transannular patch |
| 12 | 54 | M | Present | type 1 | 22 | 181 | 141 | 5 | 14 | 46 | PVR |
| 13 | 59 | F | Present | type 1 | 50 | 122 | 61 | 7 | 12 | 41 | Transannular patch |
| 14 | 29 | F | Present | type 3 | 63 | 102 | 38 | 49 | 18 | 46 | Patch infundibuloplasty |
| 15 | 38 | M | Present | type 3 | 66 | 73 | 24 | 5 | NA | 40 | Patch infundibuloplasty |
| 16 | 24 | F | Present | type 2 | 59 | 181 | 74 | 35 | 12 | 29 | Patch infundibuloplasty |
| 17 | 29 | F | Present | type 2 | 58 | 110 | 41 | 42 | NA | 35 | Patch infundibuloplasty |
| 18 | 44 | M | Present | type 2 | 47 | 126 | 66 | 41 | NA | 71 | Muscular resection |
| 19 | 46 | F | Present | type 2 | 44 | 158 | 89 | 59 | 18 | 44 | Patch infundibuloplasty |
| 20 | 15 | F | Present | type 4 | 51 | 84 | 41 | 37 | 32 | 84 | Patch infundibuloplasty |
| Abbreviations - RV: right ventricular, EF: ejection fraction, EDVi: end-diastolic volume indexed body surface area, ESVi: end-systolic volume indexed body surface area, PRF: pulmonary regurgitant fraction, mPAP: mean pulmonary artery pressure, RVSP: right ventricular systolic pressure measured, NA: not applicable, PVR: pulmonary valve replacement. | | | | | | | | | | | |

Table S1.

Detail information of 20 patients with a repaired Tetralogy of Fallot. Case numbers are correspond with case numbers in Figure 5.
